# A Flexible Multidose GnRH Antagonist versus a Microdose Flare-Up GnRH Agonist Combined with a Flexible Multidose GnRH Antagonist Protocol in Poor Responders to IVF

**DOI:** 10.1155/2015/970163

**Published:** 2015-06-16

**Authors:** Gayem İnayet Turgay Çelik, Havva Kömür Sütçü, Yaşam Kemal Akpak, Münire Erman Akar

**Affiliations:** ^1^Department of Obstetrics and Gynaecology, Akdeniz University Faculty of Medicine, 07058 Antalya, Turkey; ^2^Department of Obstetrics and Gynaecology, Ankara Mevki Military Hospital, 06110 Ankara, Turkey

## Abstract

*Objective*. To compare the effectiveness of a flexible multidose gonadotropin-releasing hormone (GnRH) antagonist against the effectiveness of a microdose flare-up GnRH agonist combined with a flexible multidose GnRH antagonist protocol in poor responders to in vitro fertilization (IVF). *Study Design*. A retrospective study in Akdeniz University, Faculty of Medicine, Department of Obstetrics and Gynecology, IVF Center, for 131 poor responders in the intracytoplasmic sperm injection-embryo transfer (ICSI-ET) program between January 2006 and November 2012. The groups were compared to the patients' characteristics, controlled ovarian stimulation (COH) results, and laboratory results. *Results*. Combination protocol was applied to 46 patients (group 1), and a single protocol was applied to 85 patients (group 2). In group 1, the duration of the treatment was longer and the dose of FSH was higher. The cycle cancellation rate was significantly higher in group 2 (26.1% versus 38.8%). A significant difference was not observed with respect to the number and quality of oocytes and embryos or to the number of embryos transferred. There were no statistically significant differences in the hCG positivity (9.5% versus 9.4%) or the clinical pregnancy rates (7.1% versus 10.6%). *Conclusion*. The combination protocol does not provide additional efficacy.

## 1. Introduction

The treatment success of in vitro fertilization (IVF) is based on various factors, including the number of retrieved oocytes [1]. Failure to recruit adequate follicles, from which the oocytes are retrieved, is called a “poor response.” The incidence of poor ovarian response (POR) in controlled ovarian hyperstimulation (COH) has been reported in 9–24% of intracytoplasmic sperm injection-embryo transfer (ICSI-ET) cycles [2]. Unfortunately, there is still no achieved consensus on the COH of a patient with POR about appropriate protocol. Insufficient follicular development with standard-dose ovarian stimulation and lower pregnancy rates are two important characteristics of POR [3]. Recently, a definition was proposed by the ESHRE Working Group on Poor Ovarian Response Definition (Bologna Criteria) to homogenize this patient population. To be classified with POR, a patient must exhibit two of the following: (1) being over the age of 40 (≥40 age) or any other risk factor for POR (pelvic infection, ovarian endometrioma, ovarian surgery, chemotherapy, and short menstrual cycle); (2) previous POR (with conventional stimulation protocol ≤ 3 oocytes); or (3) abnormal ovarian reserve test (the number of antral follicles < 5–7 or anti-Mullerian hormone (AMH) < 0.5–1.1 ng/mL) [4].

Nowadays, a microdose gonadotropin-releasing hormone (GnRH) agonist flare-up protocol combined with a flexible multidose GnRH antagonist has been used frequently in the poor responders [5–12]. This protocol (agonist-antagonist) was first introduced by Berger and his associates in 2004, yet its application remains limited [13]. In this study, we aimed to compare the effectiveness of an agonist-antagonist combined protocol with an antagonist protocol in poor responders.

## 2. Materials and Methods

This study was conducted at Akdeniz University, Faculty of Medicine, Department of Obstetrics and Gynecology, IVF Center. All of the patients in the ICSI-ET program between January 2006 and November 2012 were evaluated retrospectively. It was approved by the Ethics Committee of the Faculty of Medicine, Akdeniz University. Patients with POR who underwent COH with a flexible multidose GnRH antagonist protocol or with a flexible multidose GnRH antagonist in combination with the microdose flare-up GnRH agonist protocol were chosen for this study.

The criteria for the selection of the patients were as follows: (a) patients with ≤8 antral follicle count in bilateral ovaries and/or (b) patients with basal follicle stimulating hormone (FSH) ≥ 9 mIU/mL and/or (c) patients with a history of cancelled IVF cycle, ≤3 dominant follicles and ≤500 pmol/L estradiol (E2) levels at hCG day. The exclusion criteria were considered as follows: (a) patients over 40 years of age, (b) patients with azoospermia, (c) patients with previous ovarian surgery, and (d) patients with severe endometriosis. The criteria for cycle cancellation were as follows: (a) premature luteinizing hormone (LH) surge; (b) during COH, LH > 12 mIU/mL; (c) decrease in the level of E2 (more than 50% between 2 control days); (d) patients who failed to respond despite stimulation for 6 days; and (e) the inability to retrieve oocytes or the underdevelopment of the embryo.

A total of 131 patients with POR were selected. A microdose GnRH agonist flare-up combined with a flexible multidose GnRH antagonist protocol was applied to 46 patients (group 1) and a flexible multidose GnRH antagonist protocol was applied to 85 patients (group 2). The characteristics, responses to the COH protocol, and the embryological and clinical outcomes were recorded.

The initial blood E2, FSH, and LH levels were measured (at 8:30–9:00 a.m.) for all patients in the early follicular phase (2nd–4th days of the menstrual cycle) and on the same day the initial transvaginal ultrasonography (TVUSG) (Logiq 200 Pro, General Electric, South Korea) was performed (at 9:00–10:00 a.m.). In group 1, for the desired suppression of ovarian function, if the cyst was not greater than 10 mm after basal TVUSG, the patient began treatment with a GnRH agonist leuprolide acetate (1 mg vial, Lucrin, Abbott, France). Lucrin was diluted in the IVF unit (1 cc Lucrin corresponding to 10 cc SF) and was applied in both the morning and evening (2 × 40 *µ*g) subcutaneously (SC). After 2 days, stimulation with a high dose (300–600 IU; average 450 IU) of gonadotropin began. The dose and follow-up were individualized according to the ovarian response. The E2 and LH follow-ups were conducted together. For stimulation, recombinant-follicle stimulating hormone (R-FSH) (Puregon, Organon, Netherlands, or Gonal-F, Serono, Italy) and/or urinary-follicle stimulating hormone (u-FSH) (Menogon, Ferring, Switzerland) was used. When gonadotropin treatment began, the dose of Lucrin was reduced by half (40 *μ*g, evening) until the GnRH antagonist treatment began. When the dominant follicle was ≥12 mm and the E2 level was ≤400, treatment began with the GnRH antagonist Cetrorelix (0.25 mg vial, Cetrotide, Serono, Germany) 1 × 1 SC or Ganirelix (0.25 mg vial, Orgalutran, Organon, Netherlands) 1 × 1 SC. At the start of treatment with the GnRH antagonist, the treatment with the GnRH agonist ceased. The GnRH antagonist was continued until the day of the hCG injection. In group 2, treatment with high-dose gonadotropin (300–600 IU; mean 450 IU) began in the early follicular phase (2–4 days). When the dominant follicle was ≥12 mm and the E2 level was ≤400, treatment with the GnRH antagonist Cetrorelix 1 × 1 SC or Ganirelix 1 × 1 SC commenced.

In both protocols, when a follicle of 18 mm was observed, ovulation was triggered with human choriogonadotropin (hCG) alpha (Ovitrelle 250 mcg SC, Serono, Germany) or 10,000 IU of urinary hCG (Pregnyl 5000 IU SC freeze-dried ampoule, MSD, Baxter Pharmaceutical Solutions LLC, Bloomington, USA). Oocyte pick-up (OPU) was performed 36 hours after the hCG administration. Oocyte fertilization was performed by ICSI. In cases of successful fertilization, embryo transfer was performed on day 2 or 3 for all of the patients. All patients received luteal phase support with 2 × 90 mg of vaginal progesterone (Crinone gel 8%, Serono, UK) with weekly transdermal estradiol (Climara patch 12.5 cm^2^/3.9 mg/1 × 4 patch, Schering, Germany). If pregnancy occurred, vaginal progesterone support was continued until the 12th gestational week.

Analyses were conducted at Akdeniz University, Department of Biostatistics, using the SPSS 18.0 Package Program. Descriptive statistics are reported as frequencies, percentages, medians, and minimum and maximum values. To test whether the distributions of the parameters were normal, Kolmogorov-Smirnov and Shapiro-Wilk tests were performed. Moreover, the Mann-Whitney *U* test was used for quantitative variables, and Fisher's exact test and Pearson chi-square tests were conducted on qualitative variables. The significance level was set as 0.05, and values less than 0.05 were considered statistically significant (*p* < 0.05).

## 3. Results

The patients' characteristics are presented in Table 1. As shown, patients in group 1 exhibited significantly higher day 3 FSH levels (*p* = 0.001), whereas the duration of infertility (*p* = 0.036) and the number of previously canceled cycles were significantly higher in group 2 (*p* = 0.032).

The analysis of the COH results revealed that the duration of the stimulation was longer (*p* = 0.003), the dose of FSH was higher (*p* = 0.016), and the antagonist was started earlier (*p* = 0.001) in group 1 than in group 2 (Table 2).

Unlike the laboratory results, there was no significant difference with respect to the number and quality of oocytes and embryos obtained and transferred (Table 3). In addition, there were no statistically significant differences in the cancellation rates (26.1% for group 1, 38.8% for group 2) (*p* = 0.143), hCG positivity (9.5% for group 1, 9.4% for group 2) (*p* = 0.984), or clinical pregnancy rates (7.1% for group 1, 10.6% for group 2) (*p* = 0.532).

## 4. Discussion

There is no existing standard COH protocol that is preferred in ICSI-ET for poor responders. There are several studies related to GnRH antagonists that are commonly used for patients with POR [5–11]. The aim of antagonist application is to prevent premature LH output and suppress the effect of GnRH analogues on the ovaries so the maximum cohort of ovarian oocytes can be attained [14, 15]. Albano et al. [16] investigated the early and midluteal LH concentrations in cycles in which human menopausal gonadotropin (HMG) was applied with the addition of antagonist. The authors observed that LH could be controlled in the group with additional antagonist. Lin et al. [17] compared agonists and antagonists. In an in vitro cell culture environment, the IVF granulosa cells from patients in the antagonist group inhibited less of the agonist and were there reported to be superior in terms of progesterone secretion. However, the long-term use of GnRH agonists in patients with POR leads to an increase in cancellation rates due to the suppression of ovarian response. Although there was no significant difference in the number of mature oocytes obtained, the duration of the treatment and follow-up were extended; the cost would thus increase. The use of GnRH analogues in the luteal phase with a long protocol not only supplies ovarian suppression, which is necessary for synchronizing ovarian stimulation, but also prevents restimulation in patients with POR. In their prospective, nonrandomized study, Surrey et al. [12] reported that, for 34 patients with POR induced by long protocol and 34 patients for whom IVF failed, a microdose flare-up protocol with a GnRH agonist (leuprolide 80 micrograms/day SC) resulted in an increase in the clinical pregnancy rates. Scott and Navot [18] observed higher E2 levels and more oocytes using even lower doses (20 *µ*g) of leuprolide. However, other studies have reported conflicting results [19]. Faber et al. [20] reported promising results with the short-term discontinuation of the analogue after ovarian suppression. In this instance, the biggest disadvantage is the necessity of consuming the gonadotropin bulb. Garcia-Velasco et al. [21] reported that, after implementing a prospective randomized study, a greater number of mature oocytes could be obtained with the early withdrawal of the analogue. Additionally, similar pregnancy rates could be achieved using the long protocol. The authors found that fewer gonadotropin ampoules were used, whereas the cancellation, pregnancy, and implantation rates were similar.

The ideal approach for patients who do not respond well to conventional COH remains unknown. This study used the flexible multiple-dose antagonist protocol, which is an ideal protocol for patients with poor responses, and the more untested “agonist-antagonist protocol.” The cancellation rates were 26.1% for group 1 and 38.8% for group 2. However, no significant difference was observed between the groups (*p* = 0.143). The clinical pregnancy rates were 7.1% for group 1 and 10.6% for group 2, and the difference between the groups was insignificant (*p* = 0.532). Moreover, we observed a significant positive difference in the FSH level on day 3 in the group where agonist-antagonist protocol was used. Again, this protocol significantly increased the duration of the treatment, elevated the FSH dose, and decreased the time of starting the antagonists. For all other variables, no significant differences were observed between the groups. These results were expected because the sample consisted of patients with poor ovarian responses. The lack of a desensitization period and a lower incidence of ovarian hyperstimulation syndrome (OHSS) are due to the gonadotropin doses and the stimulation period. Therefore, antagonists may be a less cost-effective treatment, depending on the physical side effects of the agonists and antagonists and the similar reported rates of pregnancy [22]. Despite the significantly high FSH levels on day 3 and the higher rates of cancellation observed in the antagonist group, similar pregnancy rates were obtained in both groups.

The increase in the ovarian response can be explained by several mechanisms. First, pituitary suppression with GnRH analogues prevents a premature LH rise, so the rate of cycle cancellation is reduced. However, this may result in gonadotropin suppression at higher rates. Conversely, the use of GnRH analogues and the downregulation of gonadotropin partially inhibit the regulation of ovarian steroidogenesis and oocyte maturation. It is suggested that GnRH analogues have a direct inhibitory effect on ovaries. Therefore, decreasing the dose of GnRH analogues or completely ceasing treatment increases the ovarian response [23]. This hypothesis was based on the fact that there are GnRH receptors on the ovaries [24]. However, a study by Aleem and Predanic [25], based on the analysis of Doppler, indicated that GnRH analogues reduce blood flow. To ensure follicular development, it is necessary that gonadotropins reach the ovaries by a well-functioning vascular bed. Based on these data, it is believed that pituitary suppression can be achieved by stopping GnRH analogues early. However, perifollicular blood flow, which is decreased after stopping analogue treatment, can retain the analogue. The number of oocytes obtained and the subsequent IVF results are directly associated with perifollicular blood flow.

The objective of a short or flare protocol is to increase the effectiveness of exogenous hormones using the inflammatory effect of endogenous gonadotropins of GnRH analogues. The objective of agonist protocols is not only to synchronize the follicular development but also to prevent premature LH output. The microdose flare-up protocol is the preferred protocol and is used successfully in patients who are poor responders. The advantage of this approach is that, in the early follicular phase, adding low-dose GnRHa initially and providing endogenous gonadotropin secretion (flare effect) after the addition of exogenous gonadotropins increase the response [12, 18]. This flare effect may cause premature luteinization while increasing follicular recruitment [26]. The disadvantage of this approach is that, during the early follicular maturation, increasing the levels of progesterone and testosterone using serum LH may affect the quality of the oocytes [27].

In our study, we used a microdose GnRH agonist flare-up combined with a flexible multidose GnRH antagonist protocol, which is a rare application in the literature. Follicular “recruitment” takes place in the late luteal and early follicular phases. In patients with POR, excessive suppression by a high dose of GnRHa was avoided, and the flare effect of low-dose GnRHa was induced for a short time. Moreover, we tried to prevent the possible LH peak by starting the GnRH antagonist and stopping the GnRHa at the right time. In the protocols used by both groups in our study, the preoral contraceptives (OCPs) application was not used as a standard. A basal ultrasound was used in patients with OCPs in the presence of cysts over 12 mm. Duvan et al. [28] reported that, among the patients with poor response who were treated with a microdose flare-up protocol, there was no significant difference in the number of oocytes, the peak E2 levels, the endometrial thickness, the fertilization rates, or the embryo quality between the groups with or without OCPs. By minimizing the harmful effects of these two protocols for patients with poor responses, the beneficial effects of the combination of microdose flare-up and GnRH were applied for the first time by Berger et al. [13]. The new protocol was defined as the “agonist-antagonist protocol.” Berger et al. reported a 13% clinical pregnancy rate with this combination. With this protocol, the sudden flare effect suppressed endogenous FSH or was combined with the effect achieved by a GnRH antagonist. Similarly, Orvieto et al. [29] treated 21 patients who previously exhibited weak responses to IVF cycles (≤5 oocytes previous cycles) with a very short GnRH agonist and a flare flexible multidose GnRH antagonist. Orvieto et al. administered the same protocol to 10 patients in 2009 with the secondary aim of examining embryo quality. High-quality embryos were obtained significantly more frequently, and these results were associated with higher pregnancy rates (50%). Orvieto et al. [30] reported that, in the control group, the results were not satisfactory due to the patients' previous unsuccessful cycles. Berker et al. applied an ultrashort GnRH agonist-antagonist protocol to 41 patients. A microdose flare-up protocol was administered to another 41 patients. In both groups, a similar cycle cancellation rate was observed. Similar to our study, the stimulation time and the total dose of gonadotropin were significantly higher in the agonist-antagonist protocol. Other variables were not statistically significant. Although the pregnancy rate (hCG positivity) was higher in the microdose flare-up protocol, the difference was not statistically significant (26.3% versus 19.5%) [31].

## 5. Limitations of the Study

Body mass index (BMI) data were excluded from the study because we could not achieve reaching all patients' data. If they would be included in the study, quality of the results could be improved. The significant differences between two specific groups regard to above a certain level of basal FSH presumably may not affect the results of our study because of including only poor responder patients in the study.

## 6. Conclusion

As a result, the results obtained using a flexible multidose antagonist protocol were similar to those obtained using the standard protocol. This suggests that the new combination may be preferred in patients with poor responses in the future. However, larger scale and prospective studies are needed for more precise results. This combination treatment, which is rarely observed in the literature, requires a larger sample size to validate its utility.

## Figures and Tables

**Table 1:** Characteristics of patients.

	Group 1^*∗*^ (*n* = 46)	Group 2^*∗∗*^ (*n* = 85)	*p* value
Age (years)	36.5 (26–40)	35 (26–40)	0.865
Duration of infertility (years)	5.5 (0.5–20)	9 (0.5–22)	**0.036**
Day 3 FSH level (mIU/mL)	14.4 (7–22)	11 (3.4–29)	**0.001**
Day 3 E2 level (pg/mL)	39.1 (6–126)	36 (14–148)	0.684
The number of antral follicles on day 3	2 (0–8)	1 (0–8)	0.869
The number of previous cycles	0 (0–6)	0 (0–4)	0.516
The number of previously canceled cycles	0 (0–3)	0 (0–3)	**0.586**

The values are given as the medians (minimum–maximum).

^*∗*^A microdose GnRH agonist flare-up combined with a flexible multidose GnRH antagonist protocol.

^*∗∗*^A flexible multidose GnRH antagonist protocol.

**Table 2:** The comparison of the COH results.

	Group 1^*∗*^ (*n* = 46)	Group 2^*∗∗*^ (*n* = 85)	*p* value
Duration of the treatment (days)	12 (7–30)	11 (7–16)	**0.003**
Dose of FSH (IU)	4200 (750–12600)	3375 (0–8550)	**0.016**
Total dose of HMG (IU)	562,5 (0–4500)	450 (0–4200)	0.397
Starting time of antagonist (day)	7 (0–11)	9 (5–13)	**0.001**
E2 level on hCG day (pg/mL)	1129 (224–10840)	1206 (219–9570)	0.186
Number of antral follicles at USG on hCG day	3 (0–7)	3 (1–12)	0.640

The values are given as the medians (minimum–maximum).

^*∗*^A microdose GnRH agonist flare-up combined with a flexible multidose GnRH antagonist protocol.

^*∗∗*^A flexible multidose GnRH antagonist protocol.

**Table 3:** The laboratory results of the two groups.

	Group 1 (*n* = 46)	Group 2 (*n* = 85)	*p* value
The number of oocytes	1 (0–8)	2 (0–8)	0.097
M1 oocytes	0 (0–2)	0 (0–4)	0.413
M2 oocytes	1 (0–6)	1 (0–6)	0.277
P1 oocytes	0 (0–2)	0 (0–3)	0.950
Degenerate oocytes	0 (0-1)	0 (0-1)	0.760
The number of embryos	1 (0–4)	1 (0–5)	0.613
G1	0 (0–3)	0 (0–4)	0.920
G2	0 (0–3)	0 (0–3)	0.276
G3	0 (0-1)	0 (0–2)	0.063
The number of embryos transferred	1 (0–2)	1 (0–2)	0.960
